# Evidence of Disseminated Intravascular Coagulation in a Hemorrhagic Fever with Renal Syndrome—Scoring Models and Severe Illness

**DOI:** 10.1371/journal.pone.0021134

**Published:** 2011-06-23

**Authors:** Erik Sundberg, Johan Hultdin, Sofie Nilsson, Clas Ahlm

**Affiliations:** 1 Department of Clinical Microbiology/Infectious Diseases, Umeå University, Umeå, Sweden; 2 Department of Medical Biosciences/Clinical Chemistry, Umeå University, Umeå, Sweden; Leiden University Medical Center, Netherlands

## Abstract

**Background:**

Viral hemorrhagic fevers (VHF) are considered to be a serious threat to public health worldwide with up to 100 million cases annually. The general hypothesis is that disseminated intravascular coagulation (DIC) is an important part of the pathogenesis. The study objectives were to study the variability of DIC in consecutive patients with acute hemorrhagic fever with renal syndrome (HFRS), and to evaluate if different established DIC-scores can be used as a prognostic marker for a more severe illness.

**Method and Findings:**

In a prospective study 2006–2008, data from 106 patients with confirmed HFRS were analyzed and scored for the presence of DIC according to six different templates based on criteria from the International Society on Thrombosis and Haemostasis (ISTH). The DIC-scoring templates with a fibrinogen/CRP-ratio were most predictive, with predictions for moderate/severe illness (p<0.01) and bleeding of moderate/major importance (p<0.05). With these templates, 18.9–28.3% of the patients were diagnosed with DIC.

**Conclusions:**

DIC was found in about one fourth of the patients and correlated with a more severe disease. This supports that DIC is an important part of the pathogenesis in HFRS.

ISTH-scores including fibrinogen/CRP-ratio outperform models without. The high negative predictive value could be a valuable tool for the clinician. We also believe that our findings could be relevant for other VHFs.

## Introduction

The term viral hemorrhagic fever (VHF) constitutes disease caused by a diverse group of highly pathogenic RNA viruses from four taxonomic families: *Arenaviridae*, *Bunyaviridae*, *Filoviridae* and *Flaviviridae* [1], [2]. As a group, VHFs are annually estimated to cause 50–100 million cases of illness of various severity worldwide [3]. Dengue virus *(Flaviviridae)*, is responsible for the absolute majority of these cases [4]. Most VHFs are arthropod borne zoonoses and have, due to e.g. climate changes and land use, been reported as emerging. This poses a serious threat to public health in endemic areas [4]. Common features for VHFs are fever, malaise and a propensity for bleeding, thrombosis and shock [5]. The severity of VHF infections varies within the extremes. Case-fatality rates in up to 90% are shown among those infected with Ebola and Marburg virus (*Filoviridae*) [6], [7]. Hantaviruses *(Bunyaviridae)* are carried by rodents and transmission to humans is caused by inhalation of infected rodent excreta. Hantaviral infections cause two different febrile illnesses in human, hemorrhagic fever with renal syndrome (HFRS) in Eurasia and the more severe hantavirus cardiopulmonary syndrome in the Americas. It has been estimated that 250.000–300.000 cases occur annually [8]. Depending on the causative virus, different hantaviruses have a reported mortality varying between 0.5–40% [8], [9], [10], [11].

Puumala virus, genus *Hantavirus*, is the causative agent for the HFRS in the present study. This virus is endemic in Europe and causes a mild form of HFRS, also denoted nephropathia epidemica (NE) which has a lower mortality [10], [12]. Besides symptoms mentioned for VHFs as a group, HFRS commonly show renal impairment and transient thrombocytopenia is often diagnostic for the disease [13], [14]. Mechanisms for thrombocytopenia has been discussed, such as interaction between hantavirus and platelets or hantavirus and megakaryocytes [9], [15], [16]. Bleeding of moderate/major importance has been reported in up 5% of the patients infected with Puumala hantavirus [13]. The mechanisms behind the pathogenesis of acute VHF are complex and partly unknown. The ability to infect endothelial cells is considered a key role in developing HFRS and the hallmark of hantaviral diseases is altered vascular function and capillary leakage, ultimately leading to fatal hypotensive shock in severe cases [9], [17], [18]. Infection of the endothelial cells is suggested to cause some tissue damage [19], but unlikely to be the primary cause of pathology since Hantaviruses are not cytopathic in vivo [20]. In addition, inflammation normally shifts the haemostatic system in favor of thrombosis by multiple mechanisms [21]. These changes in coagulation may range from limited laboratory deviations to disseminated intravascular coagulation (DIC), a syndrome with various degree of microvascular thrombi, thrombocytopenia, and hemorrhage that may cause failure of the microvasculature and contribute to organ dysfunction [22], [23]. It has earlier been discussed that the bleeding diathesis in VHF patients may be caused by defect platelet function and DIC has been observed [24], [25], [26]. Similarly, earlier reports suggest that some patients with HFRS present signs of DIC [27], [28], [29], [30], [31], [32], [33], [34]. Today the treatment of HFRS is symptomatic with dialysis in some selected cases and as for most VHFs, no useful prognostic marker for severe infection is available at present [5], [9].

To standardize and facilitate the diagnosis of DIC by a practical, reproducible and flexible diagnostic criteria, a committee within the International Society on Thrombosis and Haemostasis (ISTH) developed two separate scoring systems in 2001 [35]. These scoring templates are based on a combination of readily available laboratory coagulation tests and were designed to define the two proposed delinineated phases of the condition, i.e. non-*overt DIC*, with haemostatic dysfunction but still a compensated coagulation system, and *overt DIC*, with a decompensated coagulation system. Earlier studies have shown the overt DIC template to have high sensitivity and specificity for DIC [36], and also that replacing fibrinogen with a fibrinogen/CRP-ratio further enhanced the diagnostic and prognostic power of the template [37]. Recently, Laine et al. showed that 5 out of 19 patients with NE fulfilled the criteria for DIC according to ISTH [38].

In a recent, large outbreak of HFRS in northern Sweden [39] we experienced that several patients became critically ill with thromboembolic complications and/or life threatening bleedings. Our hypotheses were that these patients meet international criteria for DIC, and that the presence of DIC could be used as a prognostic marker for disease severity.

## Materials and Methods

### Ethics Statement

The study was approved by the Research Ethics Committee of Umeå University, Umeå, Sweden. Informed written consent was obtained from all patients.

### Subjects

Patients (n = 119) with confirmed HFRS, either admitted to the Clinic of Infectious Diseases at Umeå University Hospital or seen by the on-call doctor and later followed policlinically, were included consecutively during September 2006 to June 2008. The diagnosis of HFRS was based upon typical clinical manifestations, e.g. fever, malaise, thrombocytopenia, and proteinuria, and confirmed by Puumala virus specific IgM and IgG antibodies as detected by immunofluorescence assay. Patients admitted late in the course of infection or patients with missing laboratory data that could not be scored for the presence of DIC within 12 days from onset of symptoms were excluded (n = 13). After exclusion, data from 106 patients were available for further analysis. The median age of the subjects was 53 y (range 20–83 y) and 58 (54.7%) were female.

### Criteria for moderate/severe illness

Patients were divided into two groups; mild respectively moderate/severe illness. Patients meeting two or more of the following criteria were considered to have a moderate/severe illness: treated in the intensive care unit (ICU), dialysis, radiologically verified thrombosis, need for platelet transfusion, moderate/severe hypotension (systolic blood pressure ≤90 mmHg and intravenous fluid treatment at any given time during hospitalization), and bleeding of moderate/major importance, i.e. gastrointestinal bleeding, macroscopic hematuria, and metrorrhagia.

### DIC-scoring according to ISTH

All patients were scored on days with sufficient laboratory data and the presence of overt and non-overt DIC was defined as a score of ≥5 points [35], [40]. The non-overt DIC scoring template uses both major criteria; abnormal levels of fibrin degradation products (D-dimer), platelet count, prothrombin time (PT), and minor criteria; e.g. antithrombin and Protein-C. The non-overt DIC scoring template focuses on repetitive tests and trends to facilitate early intervention. When using only the major criteria for non-overt DIC, sufficient robustness was achieved for detecting hemostatic dysfunction that had a prognostic significance in unselected severely ill patients [40]. In addition to the major criteria used in the non-overt DIC template, the overt DIC template also uses fibrinogen, an acute phase reactant that is almost always elevated in patients with infection and/or inflammation [41]. The ISTH scoring table has set values for platelet count, PT and fibrinogen but uses “moderately” and “strongly” increased values for D-dimer. The original ISTH overt DIC-scoring template allows a choice of two different models based on D-dimer cut-offs [42]. Correction for fibrinogen/CRP-ratio has been suggested and was included [37]. This gave us four different overt DIC templates (DIC 1–4, Table 1). DIC 1 and 3 were variants which used the local decision limit for D-dimer (0.2 mg/L) for “moderately”, and ten times this value (2.0 mg/L) for “strongly” elevated D-dimer [43]. Cut-offs for DIC 2 and 4 were based on all D-dimer tests taken in the ICU over 20 months (n = 3980). The 25^th^ and 75^th^ percentiles (0.64 and 2.48 mg/L) were used for moderately and strongly elevated D-dimer in these templates [44]. PT was converted to PK-INR in our scoring. The same cut-offs for D-dimer and PK was applied when modifying the original non-overt DIC template into 2 non-overt DIC templates (NO DIC 1 and 2, Table 1).

**Table 1:** Scoring templates for overt- and non-overt (NO) disseminated intravascular coagulation (DIC) in patients with hemorrhagic fever with renal syndrome.

	Score	DIC1	DIC2	DIC3^a^	DIC4^b^	Score	NO-DIC1	NO-DIC2
**Platelet count, 10^9^/L**	2	<50	<50	<50	<50	1	<100	
	1	50–100	50–100	50–100	50–100	0	>100	
	0	>100	>100	>100	>100			
**D-dimer, mg/L**	3	>2.0	>2.48	>2.0	>2.48	1	≥0.2	≥0.64
	2	0.2–2.0	0.64–2.48	0.2–2.0	0.64–2.48	0	<0.2	<0.64
	0	<0.2	<0.64	<0.2	<0.64			
**PK-INR** c	2	>1.4	>1.4	>1.4	>1.4	1	≥1.2	
	1	1.2–1.4	1.2–1.4	1.2–1.4	1.2–1.4	0	<1.2	
	0	<1.2	<1.2	<1.2	<1.2			
**Fibrinogen, g/L**	1	<1.0	<1.0					
	0	≥1.0	≥1.0					
**Fibrinogen/CRP-ratio**	1			<104	<104	Falling values add 1 point and rising		
	0			≥104	≥104	values subtract 1 point for PK and		
						D-dimer (vice versa for platelets)		

DIC1 and NO-DIC1 correspond to standard ISTH scoring using local decision limit. DIC—2 and NO-DIC-2 uses D-dimer cut-offs based on ICU-patients. DIC3-4 are corrected with a fibrinogen/CRP-ratio instead of fibrinogen.

The original ISTH SSC template uses “moderately” and “strongly” increased values for D-dimer cutoffs [35]. In this table laboratory cutoffs are set from local decision limits and also corrected with a fibrinogen/CRP ratio [37]. Overt DIC-score ≥5p: compatible with overt DIC, <5p: suggestive for non-overt DIC.

a D-dimer cutoff as in DIC1, fibrinogen corrected with fibrinogen/CRP-ratio.

b D-dimer cutoff as in DIC2, fibrinogen corrected with fibrinogen/CRP-ratio.

c PK-INR <1.2 was equal to PT-prolongation <3 sec, a value between 1.2 and 1.4 was equal to PT-prolongation >3 but <6 sec, and values >1.4 were equal to PT-prolongation >6 sec.

### Blood Samples and Assays

Peripheral blood was drawn in commercially available vacutainers (Becton Dickinson, Franklin Lakes, NJ, USA). Platelet counts were performed on EDTA-anticoagulated blood on a hematology analyzer, XE-2100, and blood collected in sodium citrated tubes was analyzed for D-dimer, fibrinogen and PK-INR on a CA7000 (both Sysmex, Kobe, Japan). Creatinine and C-reactive protein (CRP) were measured on blood collected in serum separator test tubes on a Vitros 5.1 automated analyzer (Ortho-Clinical Diagnostics, Inc., Rochester, NY, USA). Puumala virus specific IgM and IgG antibodies were measured in serum using an immunofluorescence assay as previously described [45].

### Statistical Analysis

Statistical analyses were performed using SPSS for Windows, version 16.0 (SPSS Inc., Chicago, IL, USA). Fischer exact test and χ^2^ test were used for categorical variables. Mann-Whitney U test was used for continuous non-normally distributed variables and all comparisons were unpaired. For statistical calculations the first available DIC-score, and also the highest score was used. In cases with the same DIC-scores on several occasions, the first score after onset of symptoms was used. Logistic regression models were used to determine odds ratios. Performance of the different scoring systems was tested with receiver operating characteristic curves (ROC) and quantified by calculating the area under the curve (AUC) and 95% confidence interval (CI). Two-sided p values<0.05 was considered statistical significant. Statistics are based on the highest available DIC-score unless otherwise stated.

## Results

### Patient description

Patient characteristics are summarized in Table 2. Bleeding of moderate/major importance was recorded in 8.5% of the patients. Moderate/severe illness was found in 13 patients (12.3%). These patients were older compared to those with mild illness and no gender difference was seen. The moderate/severe ill also had generally higher creatinine, D-dimer and a lower nadir platelet count. They were also all admitted to the hospital, required longer hospitalization and suffered more from bleeding of moderate/major importance. All radiologically verified venous thrombosis (n = 3), i.e. pulmonary embolism, thrombosis of the porta/mesenterica veins and thrombosis of the lower extremities, were found in this group. All cases of thrombosis were diagnosed after the acute phase of the disease. They all had a nadir platelet count <50×10^9^/L during the acute phase. One patient with thrombosis also developed bleeding of moderate/major importance. All patients who developed moderate/major bleeding had, compared to patients who did not, a nadir platelet count of <150×10^9^/L.

**Table 2:** Comparison of laboratory and clinical parameters in patients with moderate/severe vs. mild hemorrhagic fever with renal syndrome.

	All (n = 106)	Moderate/severe illness, (n = 13)	Mild illness (n = 93)	p value^a^
**Age, years** b	53 (40–64)	64 (55–74.5)	51 (38.5–51.5)	0.011
**Gender, female/male, n (%)**	58/48 (54.7/45.3)	7/6 (53.8/46.2)	51/42 (54.8/45.2)	0.946
**Laboratory data at score** c				
Platelet count, ×109/L	95 (17–404)	38 (17–268)	100 (19–404)	0.001
D-dimer, mg/L	0.70 (0.12–5.31)	0.90 (0.61–5.31)	0.66 (0.12–3.69)	0.021
PK-INR	1.1 (0.9–1.5)	1.1 (0.9–1.5)	1.0 (0.9–1.4)	0.041
Fibrinogen, g/L	4.66 (1.57–32.0)	4.02 (1.57–8.42)	4.66 (2.25–32.0)	0.137
Fibrinogen/CRP ratio	83.2 (16.4–538.6)	68.6 (28.3–162.0)	85.0 (16.4–538.6)	0.242
CRP, mg/L	57 (7–236)	56 (29–169)	58 (7–236)	0.686
Creatinine, µmol/L	133 (37–1548)	245 (67–1034)	126 (37–1548)	0.010
**Clinical data, n (%)**				
Mild bleeding	27 (25.5)	3 (23.1)	24 (25.8)	0.833
Moderate/severe bleeding	9 (8.5)	3 (23.1)	6 (6.5)	0.045
Need for intensive care	5 (4.7)	5 (38.5)	0	<0.001
Dialysis	1 (0.9)	1 (7.7)	0	0.007
Thrombosis	3 (2.8)	3 (23.1)	0	<0.001
Need for oxygen	15 (14.2)	9 (69.2)	6 (6.5)	<0.001
Thrombocyte transfusion	11 (10.4)	5 (38.5)	6 (6.5)	<0.001
Days at hospital, n^d^	3 (0–55)	11 (4–55)	3 (0–13)	<0.001
Systolic blood pressure, mmHg^e^	120 (80–180)	110 (80–160)	120 (90–180)	0.085

a Calculated using Mann-Whitney U and χ^2^ test.

b Value given as median, 25^th^ and 75^th^ percentiles within parentheses.

c Value given as median, range within parentheses.

d Value given as median, range within parentheses.

e Median value at the day of DIC-scoring (n = 88).

### DIC-scoring templates

**Figure 1:**
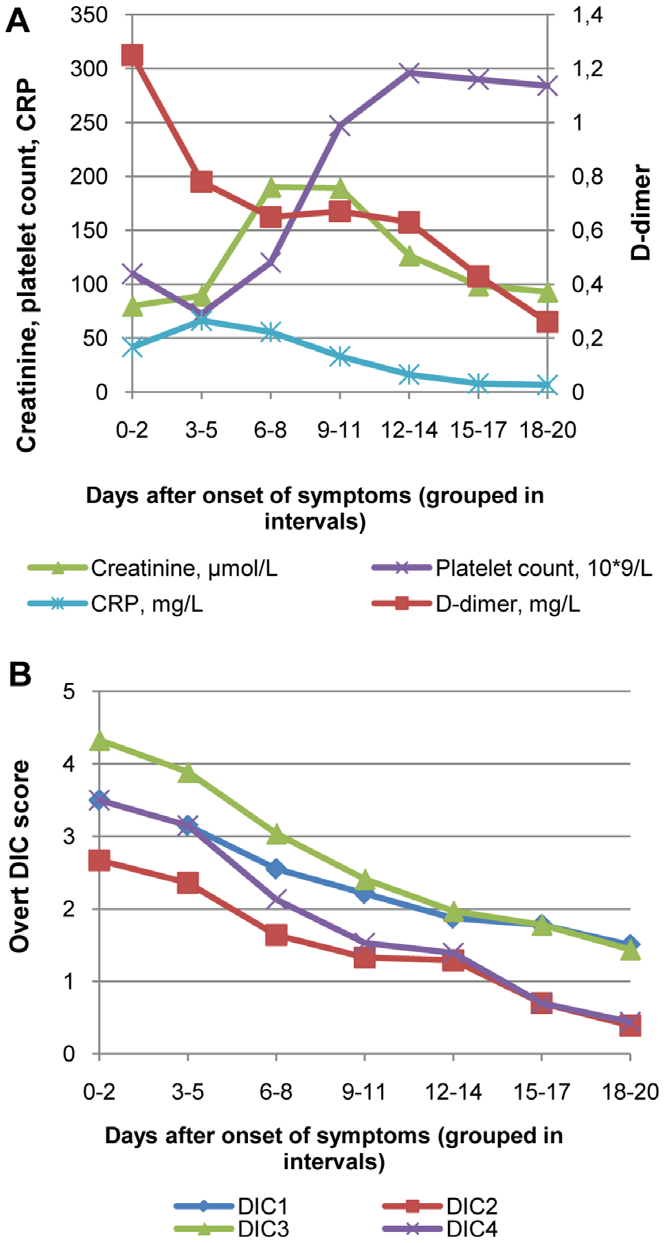
Descriptive data on 106 consecutive patients with hemorrhagic fever with renal syndrome. Figure 1a represents median laboratory data and Figure 1b represents mean overt DIC-score.

Laboratory findings and overt DIC-score kinetics during the natural course of the infection in the 106 cases are depicted in Figure 1a and 1b. The first available overt DIC-score and also the highest score both occurred on a median of day six after onset of symptoms. Depending on the template used for overt DIC (Table 1), between 3.8 to 23.6% of the patients met the criteria (≥5 points) for overt DIC with their first available score. Using the highest score during the acute phase, frequencies increased to between 5.7 to 28.3% (Table 3).

**Table 3:** Comparison between patient complications and presence of DIC according to overt- and non-overt DIC-score (≥5p and <5p respectively).

Overt DIC		n	(%)	Moderate/severe illness, n (%)	Bleeding^a^, n (%)	Intensive care, n (%)
***All***		106		13(12.3)	9(8.5)	5(4.7)
***DIC1***	≥5p	8	(7.5)	4(50.0)	4(50.0)	3(37.5)
	<5p	98	(92.5)	9(9.2)	5(5.1)	2(2.0)
	p value^b^			0.008	0.002	0.003
***DIC2***	≥5p	6	(5.7)	4(66.7)	3(50.0)	3(50.0)
	<5p	100	(94.3)	9(9.0)	6(6.0)	2(2.0)
	p value^b^			0.002	0.008	0.001
***DIC3***	≥5p	30	(28.3)	9(30.0)	6(20.0)	3(10.0)
	<5p	76	(71.7)	4(5.3)	3(3.9)	2(2.6)
	p value^b^			0.001	0.015	0.136
***DIC4***	≥5p	20	(18.9)	9(45.0)	5(25.0)	3(15.0)
	<5p	86	(81.9)	4(4.7)	4(4.7)	2(2.3)
	p value^b^			<0.001	0.011	0.045
**Non-Overt DIC**						
***All***		106		13(12.3)	9(8.5)	5(4.7)
***DIC 1***	≥5p	28	(26.4)	8(28.6)	5(17.9)	5(17.9)
	<5p	78	(73.6)	5(6.4)	4(5.1)	0
	p value^b^			0.005	0.053	0.001
***DIC 2***	≥5p	25	(23.6)	7(28.0)	5(20.0)	4(16.0)
	<5p	81	(76.4)	6(7.4)	4(4.9)	1(1.2)
	p value^b^			0.012	0.032	0.011

a Bleeding of moderate/major importance.

b Comparison between ≥5 and <5 points. P-value calculated with Fischer exact test and χ^2^ test.

Patients with ≥5 points were also more often moderate/severe ill, suffered from bleeding of moderate/severe importance, and according to overt DIC templates 1, 2 and 4 also more often required ICU-care (Table 3). Creatinine levels did not differ between the groups with and without overt DIC (data not shown). For non-overt DIC template 1 and 2, patients with a cumulative score ≥5 points were more often found to be moderate/severe ill (χ^2^-test, p<0.01, Table 3). Patients with ≥5 points in overt DIC template 3 and 4 were significantly older compared to those below (median age DIC3 49 y vs. 59 y, p = 0.034 and DIC4 49 y vs. 61 y, p = 0.011), but no differences were seen for overt DIC templates 1 and 2. No gender difference was seen for either DIC template.

The sensitivity, specificity, positive predictive value and negative predictive value of the templates for moderate/severe illness are presented in Table 4. ROC analysis of the scores for overt DIC ≥5 points compared to those below showed that only template 3 and 4 including the fibrinogen/CRP-ratio were predictive of moderate/severe illness. They also had higher negative predictive values. For non-overt DIC, both templates were predictive.

**Table 4:** Predictive values and calculated area under the curve (AUC) from receiver operating characteristic curves (ROC) of overt and non-overt DIC-templates vs. moderate/severe illness.

	Sensitivity	Specificity	PPV	NPV	AUC^a^	p-value^b^
**Overt**						
**DIC1**	30.8	95.7	50.0	91.8	0.63	0.123
**DIC2**	30.8	97.8	66.7	91.0	0.64	0.096
**DIC3**	69.2	77.4	30.0	94.7	0.73	0.007
**DIC4**	69.2	88.2	45.0	95.3	0.79	0.001
**Non-overt**						
**DIC1**	61.5	79.4	28.6	93.9	0.71	0.017
**DIC2**	53.8	81.4	28.0	92.9	0.68	0.039

Sensitivity, specificity, positive predictive value (PPV) and negative predictive value (NPV) are all presented as percentages.

a AUC, comparison between ≥5p and <5p.

b p-value for calculated AUC in predicting moderate/severe illness (ROC-analysis).

## Discussion

In this study we systematically investigated the presence of overt and non-overt DIC using internationally accepted scoring criteria in patients with confirmed HFRS. We also wanted to test if this could be used as a prognostic marker for disease severity. Our main findings are that DIC-scoring templates modified with a fibrinogen/CRP-ratio proved to be predictive and prognostic for moderate/severe illness. Using these templates, depending on the D-dimer cut off, 18.9 vs. 28.3% of the patients met the criteria for overt DIC.

In HFRS renal failure is frequent and the level of creatinine has earlier been used as a marker for clinical severity [14], [46]. A hypothesis is that in severe disease, microthrombosis in the vast microvascular beds of the kidneys, spleen, liver, and other vital organs causes organ failure. The formation of microthrombi consumes platelets and causes elevated D-dimer values, resulting in higher DIC scores. The more severely ill patients showed significantly more signs of marked capillary leakage at admission, i.e. hemoconcentration, moderate/severe hypotension and need for fluid treatment. All these findings are known complications of severe HFRS. In a recent review on symptoms, signs and severity of NE in paediatric and adult patients, the authors found severe complications such as serious internal bleeding and multi organ failure only in adult patients. Laboratory deviations such as creatinine and CRP were also significantly higher in the adults [47]. These findings are in line with ours, where patients with a more severe clinical picture were significantly older than patients with a milder clinical picture.

A low nadir platelet count and also a large drop in platelet count have earlier been shown to be an independent predictor for a poor vital outcome in an unsorted material of adult ICU patients and predict acute renal failure in nephropathia epidemica patients [14], [22], [48]. We could not confirm the relationship between a low nadir platelet count and acute renal failure in our study (data not shown).

The ISTH DIC-scoring has earlier been found useful in identifying patients with suspected DIC [37], and especially in ICU patients [40], [49], [50]. Sepsis has been proposed to be the most frequent cause of DIC with a high mortality [51]. We adjusted the templates according to local laboratory cutoff levels in line with earlier studies [42]. HFRS patients have earlier been proposed to develop DIC on a combination on clinical findings and laboratory data. The frequency of suggested DIC in these studies has ranged from a few percent up to 27% [27], [52]. The recent study by Laine et al. using the ISTH-criteria showed DIC in 26%, however this was not associated with clinical variables and failed to predict clinical outcome [38]. In the present study, up to 28.3% of the patients met criteria for overt DIC, which significantly correlated to moderate/severe illness. The difference in frequency of DIC in our study, compared to previous studies, could be explained by the earlier variations in the literature in defining the presence of DIC. Also, the ISTH DIC score is dependent on the method-specific D-dimer decision limit for exclusion of venous thromboembolism.

The ISTH overt DIC template has earlier been proposed to miss the diagnosis of early phase of overt DIC (pre-DIC) [36], [53], whereas the ISTH non-overt DIC template has been proposed to identify patients at risk for developing DIC early. Recent studies with modifications to the ISTH non-overt DIC scoring show added prediction of poor outcome in patients with sepsis with an addition of an organ system failure scoring, but also discrepancy in the usefulness of added molecular haemostatic markers such as antithrombin [51], [53]. In our study, the trend, although not significant, was that patients received their highest score somewhat earlier with the non-overt template. When using the non-overt DIC template, obvious difficulties occurred interpreting what was considered a rise or fall in laboratory data. Compared to the overt DIC templates with a fibrinogen/CRP-ratio, the non-overt DIC templates performed on par or worse with no added diagnostic value. Recent studies on unsorted patients with underlying conditions related to DIC have evaluated different modifications to the ISTH DIC scoring [36], [51], [53]. In comparison with other accepted scoring criteria for DIC, ISTH overt-DIC diagnostic criteria displayed a high specificity for DIC, was related to a poor patient outcome, and showed high number of associations with DIC in infectious disease [36]. With the added fibrinogen/CRP-ratio in our study, the sensitivity for moderate/severe illness doubled, allowing a better chance of monitoring patients at risk.

Despite ongoing studies on the use of steroids and antiviral drugs in VHF, no specific treatment is available today for HFRS. This makes a useful predictive tool for more severe illness even more valuable in finding patients who need early and aggressive symptomatic treatment. The overt-DIC scoring templates using a fibrinogen/CRP-ratio were found to identify patients at risk for developing a more severe illness. However, it is also important to prove that identifying and treating DIC improves clinical outcome. This needs to be evaluated further in larger patient samples and HFRS caused by other hantaviruses.

A limitation of this study on patients referred to the hospital is that many of them were considered to be moderate/severe ill already at presentation, i.e. before the first possible DIC score. Hence, the predictive value of the scoring systems is somewhat biased. The first and also the highest DIC score occurred on the same day in most of the cases, resulting in just slightly worse prognostic power for moderate/severe illness for the first available score. A major advantage is that the templates used are based solely on readily available coagulation tests. Thus, the templates may easily be applied in clinical routine.

In conclusion, a high proportion of patients with a mild HFRS meet the international criteria for overt DIC which increases the risk for more severe illness. It is crucial to select the best possible model for DIC-scoring. ISTH-scores including fibrinogen/CRP-ratio outperform models without. The high negative predictive value could be a valuable tool for the clinician.

We also believe that our findings could be relevant for other VHFs.
